# Construction of a focal adhesion signaling pathway-related ceRNA network in pelvic organ prolapse by transcriptome analysis

**DOI:** 10.3389/fgene.2022.996310

**Published:** 2022-09-13

**Authors:** Xia Yu, Li He, Ying Chen, Wenyi Lin, Hong Liu, Xiu Yang, Ying Ye, Xuemei Zheng, Zhenglin Yang, Yonghong Lin

**Affiliations:** ^1^ Department of Clinical Laboratory, Chengdu Women’s and Children’s Central Hospital, Sichuan Provincial People’s Hospital, School of Medicine, University of Electronic Science and Technology of China, Chengdu, Sichuan, China; ^2^ Department of Obstetrics and Gynecology, Chengdu Women’s and Children’s Central Hospital, School of Medicine, University of Electronic Science and Technology of China, Chengdu, Sichuan, China; ^3^ Department of Medical Pathology, Chengdu Women’s and Children’s Central Hospital, School of Medicine, University of Electronic Science and Technology of China, Chengdu, Sichuan, China; ^4^ Department of Surgical, Chengdu Women’s and Children’s Central Hospital, School of Medicine, University of Electronic Science and Technology of China, Chengdu, Sichuan, China; ^5^ Sichuan Provincial Key Laboratory for Human Disease Gene Study and Institute of Laboratory Medicine, Sichuan Provincial People’s Hospital, University of Electronic Science and Technology of China, Chengdu, China

**Keywords:** pelvic organ prolapse, whole transcriptome RNA sequencing, competing endogenous RNA, PPI, focal adhesion signaling pathway

## Abstract

**Objective:** Pelvic organ prolapse (POP) affects a large proportion of adult women, but the pathogenesis of POP remains unclear. The increase in global population aging will impose a substantial medical burden. Herein, we aimed to explore the related RNAs regulating the occurrence of POP and provide potential therapeutic targets.

**Method:** Tissue biopsies were collected from the anterior vaginal wall of six women with POP and six matched subjects without POP. The profiles of mRNAs, circRNAs, lncRNAs, and miRNAs were obtained by whole transcriptome RNA sequencing.

**Result:** The findings revealed that 71 circRNAs, 76 known lncRNAs, 84 miRNAs, and 931 mRNAs were significantly altered (*p* < 0.05 and |log2FC| > 1). GO and KEGG enrichment analyses indicated that the differentially expressed genes (DEGs) were mainly enriched in the focal adhesion signaling pathway. *FLT, ITGA9, VEGFD, PPP1R12B*, and *ROCK2* were identified as focal adhesion signaling pathway-related hub genes by protein–protein interaction network analysis. Based on the relationships between the DEGs and miRNA, lncRNA and circRNA targets, we constructed a focal adhesion signaling pathway-related ceRNA network. The ceRNA network includes hsa_circ_0002190/hsa_circ_0046843/lnc-CARMN -miR-23a-3p - ROCK2 and hsa_circ_0001326/hsa_circ_0007733/lnc-AC107959/lnc-TPM1-AS - miR-205-5p - ROCK2/PPP1R12B/VEGFD. Moreover, abnormalities in the cytoskeleton in fibroblasts from individuals with POP were observed.

**Conclusion:** In this study, a focal adhesion signaling pathway-related ceRNA network was constructed, and this network may serve as a target for finding suitable drugs for the treatment of POP.

## Introduction

Pelvic organ prolapse (POP) is a weakening of the pelvic floor support structure that causes the pelvic internal organs, including the bladder, rectum, and uterus, to localize into or outside the vagina, which results in a decline in quality of life and serious psychosocial problems (Collins et al., 2021; Harvey et al., 2021). According to epidemiological data, the prevalence of POP in women over the age of 60 years is as high as 50%, and the number of POP cases with clinical symptoms is expected to increase by more than 46% by 2050 (Wu et al., 2011). Anterior vaginal prolapse (AVP) is the most common form of POP. Several studies have found that vaginal wall weakness can be considered a possible cause of prolapse (Li et al., 2021). Surgery is the main treatment. However, because the pathogenesis of POP has not been fully elucidated, a possibility of reprolapse after surgery remains (Nussler et al., 2022), which results in certain difficulties in the treatment of POP.

In recent years, transcriptomics has become an important tool for exploring the pathogenesis of diseases (Wu et al., 2021). Song et al. performed RNA-seq analysis to identify the POP signatures of 81 genes in uterosacral ligament (USL) samples as well as a number of extracellular matrix (ECM)-related genes (Xie et al., 2016); but the study only examined differentially expressed (DE) genes (DEGs). In addition, many studies have examined the role of microRNAs (miRNAs) in the pathogenesis of POP. The overexpression of miRNA-30d and miRNA-181a regulates the expression of HOXA11 and collagen (Lin et al., 2020), and miRNA-92 expression may be associated with reduced estrogen receptor β1 mRNA levels in the USL of women with POP (He et al., 2016). Moreover, miR-19-3p targets IGF-1 to promote autophagy and apoptosis through the AKT/mTOR/p70s6k pathway in vaginal fibroblasts in POP (Yin et al., 2021). However, the roles of long noncoding RNAs (lncRNAs) and circular RNAs (circRNAs) in the pathogenesis of POP have not been reported. Increasing evidence shows that competing endogenous RNA (ceRNA) networks play important roles in many disease processes (Zhao et al., 2021; Liu et al., 2022). The potential roles of the circRNA/lncRNA‒miRNA–mRNA ceRNA network in the pathogenesis of POP remain unclear and have not been characterized.

Prolonged stretching and mechanical stress cause progressive deterioration of the pelvic organ support (Deng et al., 2021). Four mechanoresponsive genes are responsible for the regulation of actin cytoskeleton remodeling in fibroblasts under stretching, and investigations of the cytoskeleton phenotype in POP samples revealed an abnormal F-actin configuration (Ewies et al., 2008; Wang et al., 2015). Focal adhesion is the cell-extracellular matrix contact point that bundles actin filaments and is linked to transmembrane receptors of the integrin family *via* a multimolecular complex of junctional plaque proteins. Some focal adhesion constituents are structurally involved in the link between membrane receptors and the actin cytoskeleton, and others are signaling molecules, such as various protein kinases and phosphatases, their substrates, and adaptor proteins. These signaling events result in actin cytoskeleton reorganization. As a result, the development of a focal adhesion signaling-related ceRNA network is critical for understanding the mechanism of cytoskeletal remodeling in POP.

**FIGURE 1:**
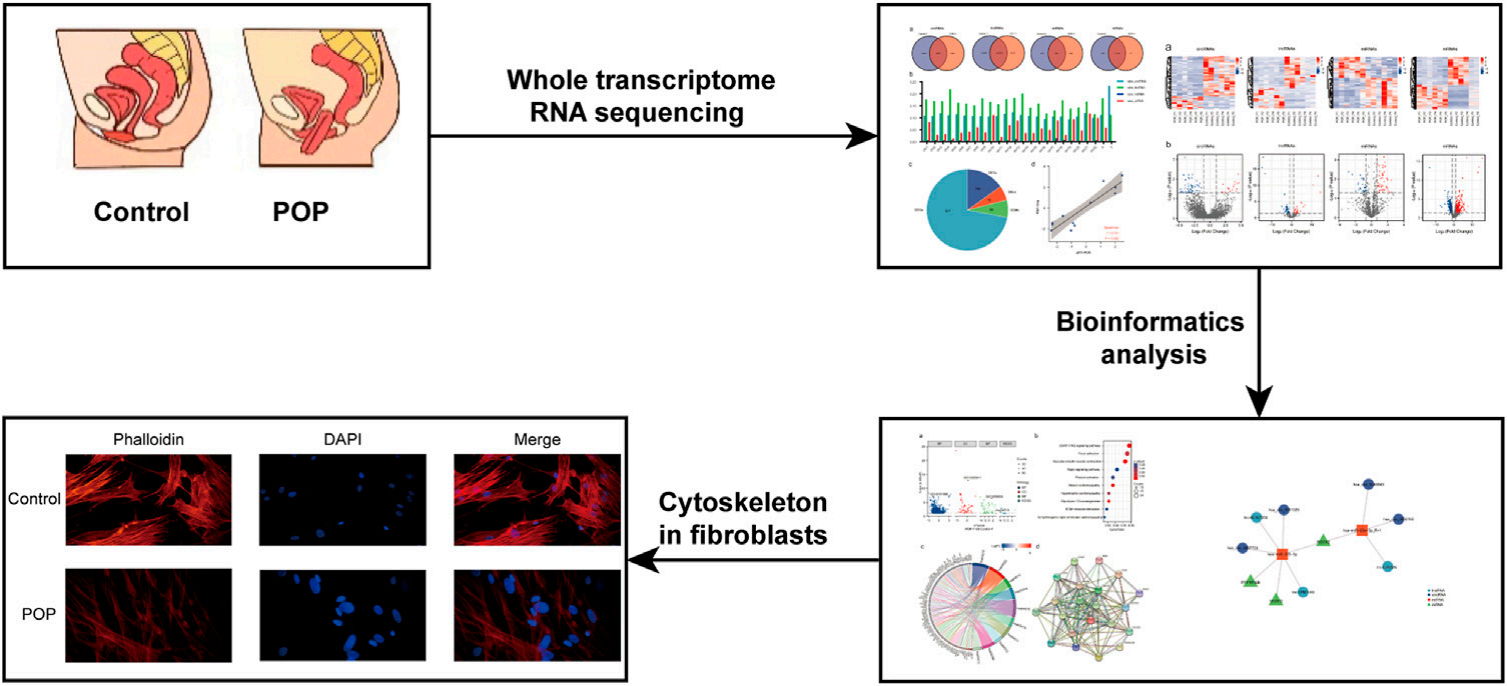
Research strategy employed in the present study.

In this study, the schematic workflow is shown in Figure 1. We designed a whole transcriptome RNA sequencing study to uncover the profiles of DE mRNAs, DEGs and ncRNAs between a POP group and a control group. Subsequently, the DEGs were evaluated by Gene Ontology (GO) and Kyoto Encyclopedia of Genes and Genomes (KEGG) pathway enrichment analyses to reveal the associated functions. Furthermore, a focal adhesion signaling pathway-related ceRNA network was constructed to explore the underlying molecular mechanisms of the ncRNAs. This study may serve as a target for finding suitable drugs to treat POP.

## Materials and methods

### Tissue sample collection

The study included 12 patients who underwent complete hysterectomy at Chengdu Women’s and Children’s Central Hospital’s Department of Obstetrics and Gynecology. The patients were screened using the following inclusion criteria: diagnosis of POP at stage 3 or higher according to the pelvic organ prolapse quantification (POP-Q) exam; patients with benign diseases (uterus myoma, adenomyosis) who did not have POP. A patient was excluded from the study if any of the following criteria applied: collagen metabolic disease or a history of hormone therapy (including vaginal estrogen therapy). Six of these patients were enrolled in the POP group, and six patients were enrolled in the control group. There were no significant differences in clinical characteristics such as age, BMI, menopausal status, gravidity, or parity between the two groups (Supplementary Table S1). The Ethics Committee of Chengdu Women’s and Children’s Central Hospital reviewed and approved this study (serial number: 2021-65), and all subjects signed informed consent forms.

After hysterectomy, 1.0 × 1.0-cm full-thickness vaginal wall tissue biopsies were obtained from the pericervical region of the anterior vaginal cuff in the controls, and from the prolapsed vaginal wall in the POP samples. Fresh tissue samples were collected aseptically, immediately frozen in liquid nitrogen, and stored at−80°C until analysis.

### Total RNA extraction

Total RNA was extracted using TRIzol reagent (Invitrogen, CA, United States) according to the manufacturer’s instructions. The RNA quantity and purity were determined using a NanoDrop ND-1000 (NanoDrop, Wilmington, DE, United States) and Bioanalyzer 2100 (Agilent, CA, United States). A small RNA library was prepared using approximately 1 µg of total RNA according to the TruSeq Small RNA Sample Prep Kit protocol (Illumina, San Diego, CA, United States). We then performed single-end sequencing (36 or 50 bp) on an Illumina HiSeq 2500 system (Illumina, San Diego, CA, United States) at LC-Bio (Hangzhou, China).

Ribosomal RNA was removed from approximately 2 µg of total RNA using the Epicenter Ribo-Zero Gold Kit (Illumina, San Diego, CA, United States) according to the manufacturer’s instructions. The purified ribosomal RNA was fragmented into small pieces using divalent cations at elevated temperatures. The cleaved RNA fragments were then reverse-transcribed to generate the final cDNA library in accordance with the mRNA-Seq sample preparation kit protocol (Invitrogen, CA, United States). The final cDNA library had an average insert size of 300 ± 50 bp. We then performed 2 × 150-bp paired-end sequencing (PE150) using an Illumina NovaSeq™ 6000 system (LC-Bio Technology Co., Ltd., Hangzhou, China).

### Bioinformatics analysis

**FIGURE 2:**
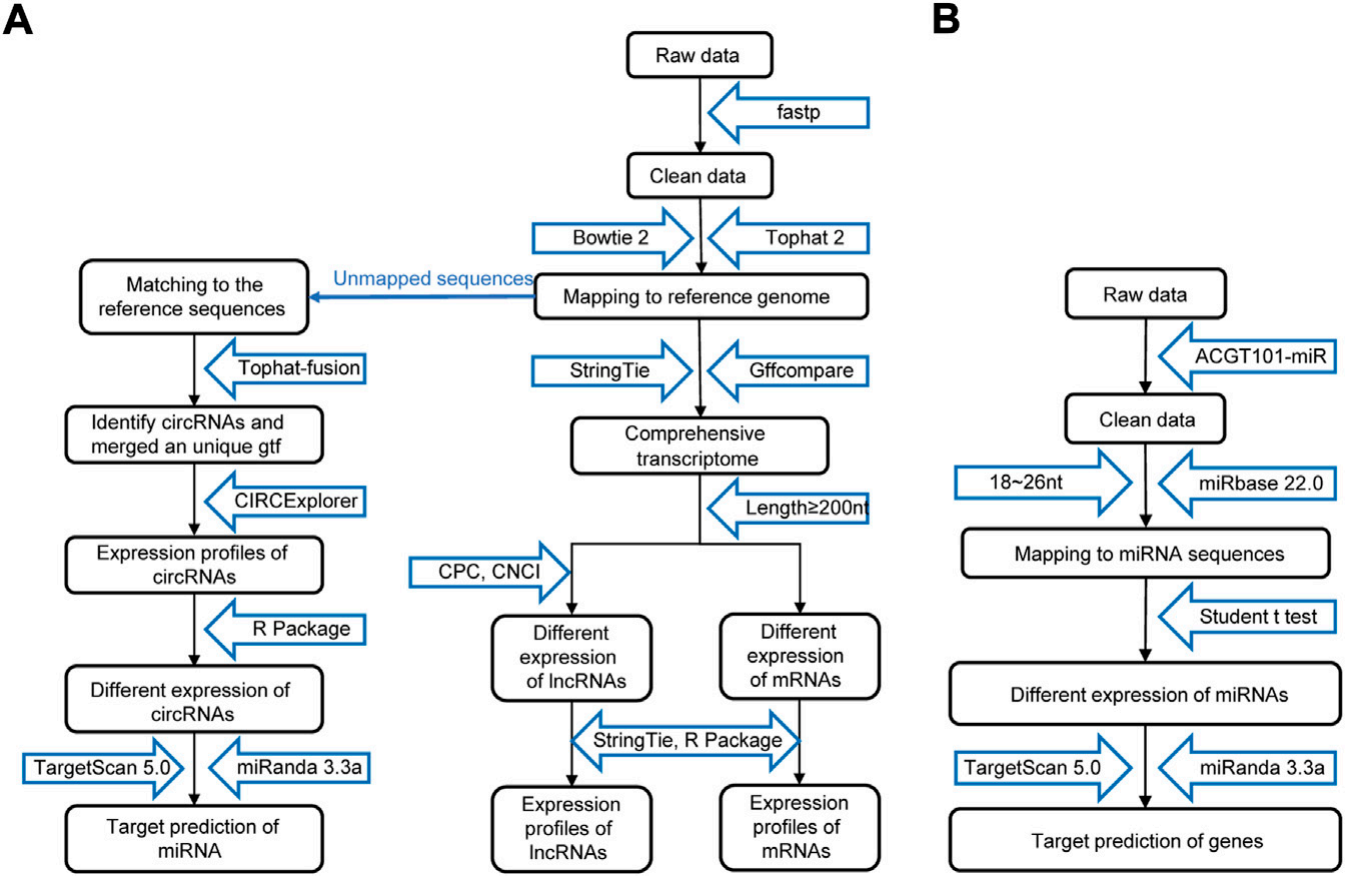
Procedure for preparing and analyzing an RNA library. **(A)** Process of preparing and analyzing circRNA, lncRNA, and mRNA libraries. **(B)** Process of preparing and analyzing miRNA libraries. CPC, coding potential calculator; CNCI, coding-noncoding index.

As shown in Figure 2A, the raw data were cleaned and verified by fastp (Chen et al., 2018), and the remaining reads were mapped to the *Homo sapiens* GRCh38 genome using Bowtie2 (Langmead and Salzberg, 2012) and Tophat2 (Kim et al., 2013) and assembled using StringTie (Pertea et al., 2015). Additionally, StringTie was used to estimate the expression levels of all transcripts. The transcripts were then annotated with known mRNAs, known lncRNAs and transcripts shorter than 200 nt were discarded, and transcripts with coding potential were predicted using CPC (Kong et al., 2007) and CNCI (Sun et al., 2013). FPKM (FPKM = [total_exon_fragments/mapped_reads (millions) × exon_length (kB)]) values showed the expression levelsof mRNAs and lncRNAs, |log2FC|>1 and *p* < 0.05 obtained using a nonpaired test comparing nested linear models were identified as DE mRNAs and lncRNAs. TopHat fusion (Kim and Salzberg, 2011) and CIRCExplorer (Zhang et al., 2014; Zhang et al., 2016) were used to identify the circRNAs. The expression of circRNAs was calculated by SRPBM = (number of back-spliced junction reads)/(number of mapped reads) × 1,000,000,000. *p* < 0.05 obtained using the R package DESeq was regarded as indicating differential expression (Anders and Huber, 2010).

The process of miRNA identification is shown in Figure 2B. A BLAST search was performed to map unique sequences of 18–26 nucleotides in length to specific species precursors in miRBase 22.0 and therefore identify known miRNAs. RNAfold software (http://rna.tbi.univie.ac.at/cgi-bin/RNAWebSuite/RNAfold.cgi) was used to predict the secondary structures of all the obtained miRNAs. A nonpaired analysis was then conducted to calculate the differential expression of miRNAs based on normalized deep-sequencing counts, and a significance threshold of 0.05 was chosen.

### RNA functional enrichment analysis

The functions of DEGs were determined through GO and KEGG analyses using the R package (v3.6.3), and *p* < 0.05 indicated a significant difference.

### Protein-protein interaction network

STRING (https://www.string-db.org) was used to build the PPI network based on the DEGs involved in the selected KEGG pathways between the POP and control groups. The extraction cutoff was set to an interaction score >0.4.

### CeRNA network

TargetScan and miRanda were used to examine the targeting relationships between DEGs, DE miRNAs (DEMs), DE lncRNAs (DELs), and DE circRNAs (DECs). The circRNA/lncRNA‒miRNA-mRNA ceRNA network was visualized using R package v3.6.3 and hub prognostic genes as the core. In the ceRNA network, this network displayed competitive binding.

### Quantitative real-time PCR analysis

qRT‒PCR analyses were performed using gene-specific primers (Supplementary Table S2). Total RNA was extracted using TRIzol reagent (TaKaRa, Kusatsu, Japan), and qRT‒PCR was performed with an Applied Biosystems StepOnePlus Real-Time PCR system (Applied Biosystems, Foster City, CA, United States) using TB Green™ Premix EX Taq™ II (TaKaRa, Kusatsu, Japan) according to the manufacturer’s instructions. Each reaction was conducted in triplicate, and the relative fold change values were measured in terms of the threshold cycle (Ct) and calculated using the formula 2^−ΔΔCt^ (Pfaffl, 2001).

### Cell culture and identification

Fibroblasts were isolated from the anterior vaginal wall. Tissues removed during hysterectomy were washed with phosphate-buffered saline (PBS) containing 1% antibiotics, cut into slices with a diameter of less than 0.1 cm, and evenly distributed in the bottom of a 25-cm bottle. To avoid floating tissue blocks, upside-down containers were used in this study, and the bottle was carefully turned over after 4–6 h. The medium contained 15% FBS (Gibco, Waltham, MA, United States), 1% penicillin/streptomycin (Gibco, Waltham, MA, United States), and DMEM (Gibco, Waltham, MA, United States). The cells were passaged after approximately 15 days. The derived cells were identified by positive immunofluorescence staining for vimentin (1:200; Bioss, Shanghai, China) and negative immunofluorescence staining for pancytokeratin (1:200; Bioss, Shanghai, China) (Supplementary Figure S1).

### Fluorescence staining of F-actin

Microscopy was used to image the fluorescently labeled F-actin network. Cells were grown on a glass coverslip in 500 µl of cell culture media to a density of 3.0 × 10^4^ cells/ml. The cells were incubated at 37°C for 48 h and then stained with TRITC phalloidin to reveal the actin cytoskeleton. The cells were fixed with 4% paraformaldehyde for 10 min, washed three times with PBS, and incubated with 0.5% Triton-X for 5 min. The cells were blocked for 30 min at room temperature in 1% BSA and then incubated for 30 min with TRITC phalloidin (1:250; Solarbio, Beijing, China) in 3% BSA. The nuclei were sealed and counterstained with DAPI. All photographs were taken with a fluorescence microscope (Shunyu, Ningbo, China) at an excitation wavelength of 540–546 nm.

## Results

### Differences in transcription files

**FIGURE 3:**
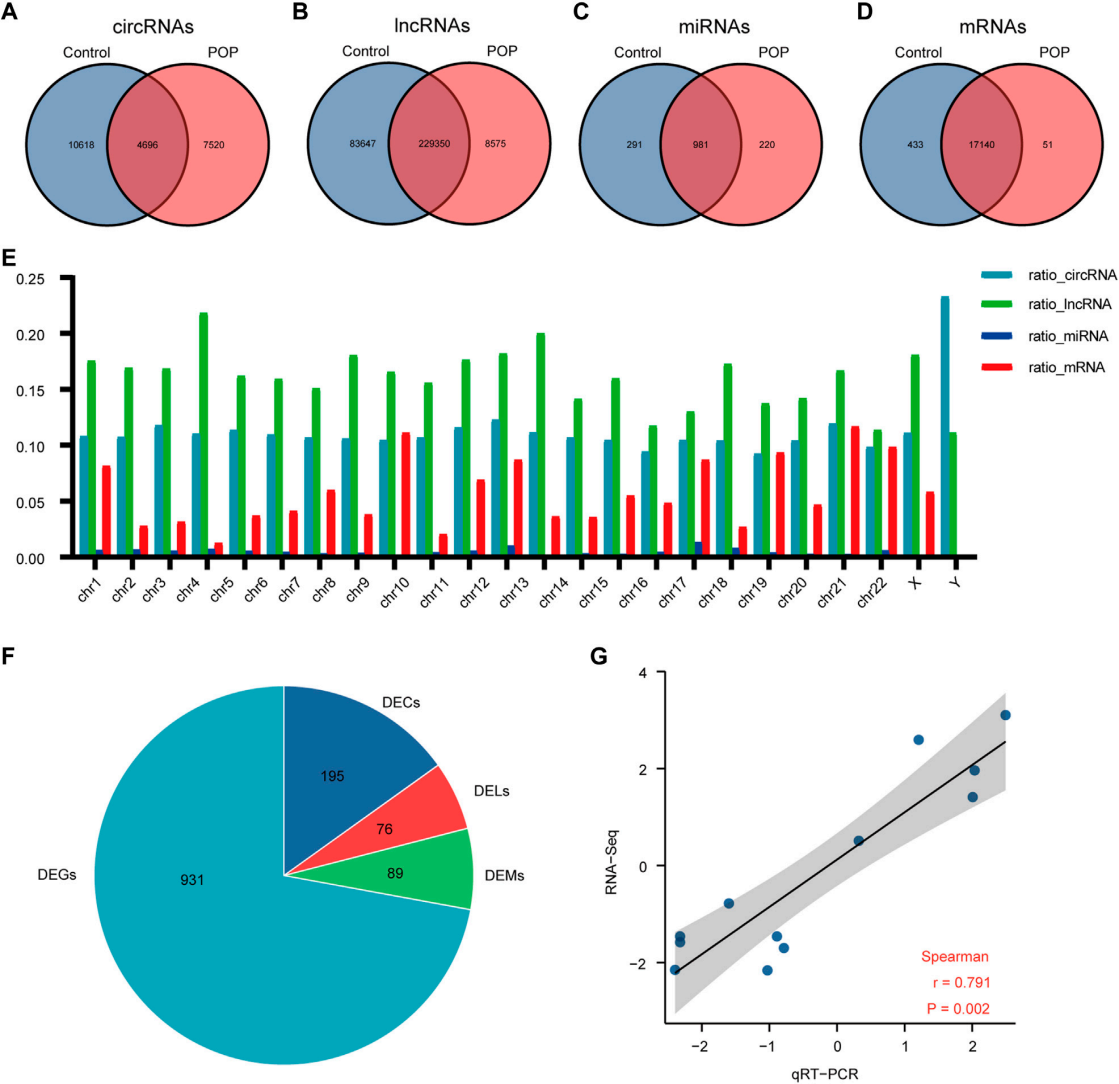
Whole transcriptome RNA sequencing results comparing the POP and control groups. **(A–D)** Venn diagrams depicting the profiles of circRNAs, lncRNAs, miRNAs, and mRNAs. **(E)** RNA expression ratio in a chromosome (number of RNAs in a related chromosome divided by the total number of RNAs). **(F)** Differentially expressed circRNAs, lncRNAs, miRNAs and mRNAs between the POP and control groups. **(G)** qRT‒PCR validation of the RNA sequencing results using 3 DECs, 3 DELs, 3 DEMs, and 3 DEGs chosen at random. The log2-fold change from the q-RT‒PCR analysis is plotted on the X-axis, and the log2-fold change from the RNA sequencing analysis is plotted on the Y-axis. POP, pelvic organ prolapse; DECs, differentially expressed circRNAs; DELs, differentially expressed lncRNAs; DEMs, differentially expressed miRNAs; DEGs, differentially expressed genes.

**FIGURE 4:**
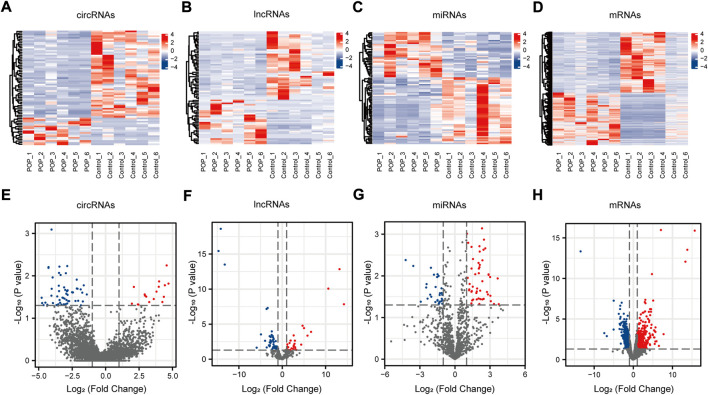
circRNA, lncRNA, miRNA, and mRNA expression profiles. **(A–D)** Heatmaps of DECs, DELs, DEMs, and DEGs in the anterior vaginal wall in the POP and control groups. **(E–H)** Volcano plots of DECs, DELs, DEMs, and DEGs in the anterior vaginal wall in the POP and control groups. POP, pelvic organ prolapse; circRNA, circular RNA; lncRNAs, long noncoding RNAs; miRNA, microRNA; DECs, differentially expressed circRNAs; DELs, differentially expressed lncRNAs; DEMs, differentially expressed miRNAs; DEGs, differentially expressed genes.

The expression profiles of circRNAs, lncRNAs, and mRNAs were determined using an Illumina NovaSeq™ 6000 system, and miRNAs were identified using an Illumina HiSeq 2500 system. After the removal of low-quality reads, the RNA-seq results revealed 22,834 circRNAs, 321,572 lncRNAs, 1,492 miRNAs, and 17,624 mRNAs (Figures 3A–D). Figure 3E depicts the chromosomal locations. Compared with the control group, 71 circRNAs, 76 known lncRNAs, 84 miRNAs, and 931 mRNAs were significantly altered in the POP group (*p* < 0.05 and |log2FC| > 1) (Figure 3F). Upregulated expression of 17 circRNAs, 30 known lncRNAs, 53 miRNAs, and 433 mRNAs and downregulated expression of 54 circRNAs, 46 known lncRNAs, 34 miRNAs, and 498 mRNAs were observed (Figures 4A–H). To validate the accuracy of our whole transcriptome data, we chose three DECs, three DELs, three DEMs, and three DEGs at random for qRT‒PCR analysis (Figure 3G). The correlation between the whole transcriptomic data and qRT‒PCR data was 0.791, indicating that our sequencing results were repeatable and dependable. The raw data can be found at https://www.ncbi.nlm.nih.gov/geo/query/acc.cgi?acc=GSE208271. The transcription levels in the anterior vaginal wall tissue differed between the POP and control groups.

### Focal adhesion is the main enrichment pathway

**FIGURE 5:**
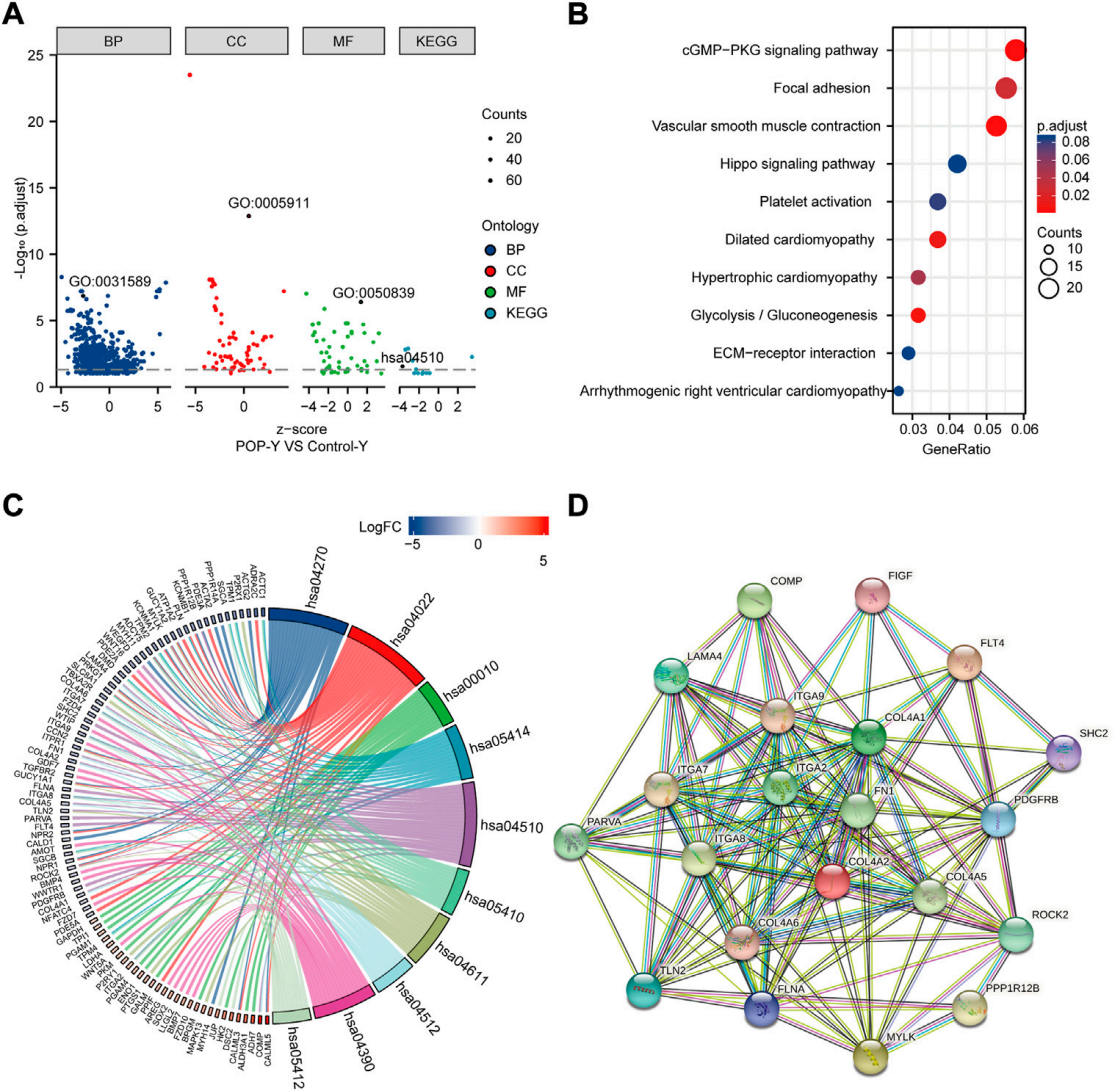
Functional enrichment analysis of DEGs using GO and KEGG and the PPI network. **(A)** Analysis of DEGs using GO and KEGG. **(B)** Top ten KEGG pathways. **(C)** Top ten KEGG pathways related to DEGs. **(D**) PPI network of focal adhesion pathway hub genes in the KEGG pathway.

GO and KEGG enrichment analyses were performed to annotate the functional and pathway roles of the DEGs. The results showed that the GO terms ‘“cell-substrate adhesion”, “collagen-containing extracellular matrix”, and “cell adhesion molecule binding” were significantly enriched in the POP group compared with the control group (Figure 5A). Figure 5B depicts the top ten enriched KEGG pathways, which included the cGMP-PKG signaling pathway, focal adhesin, vascular smooth muscle contraction and hippo signaling pathway. Furthermore, to better analyze these pathways, Figure 5C depicts the DEGs involved in the pathways. Focal adhesion is an important structure for the transmission of mechanical signals from outside to inside cells, and the KEGG enrichment analysis showed that the DEGs were significantly enriched in focal adhesion. Thus, we mainly focus on focal adhesion in the subsequent analysis.

### Hub genes that are closely related to focal adhesion signaling pathway

A PPI network was built using STRING online tools based on the DEGs associated with the focal adhesion signaling pathway (Figure 5D). Table 1 lists the enriched genes in this PPI network. We ultimately selected ITGA7, LAMA4, ROCK2, PPP1R12B, ITGA9, COL4A6, FLT4, COL4A5, COL4A2, ITGA2, VEGFD, FN1, ITGA8, and COL4A1 as hub genes because their interaction scores were >0.9.

**TABLE 1:** The enriched genes in the KEGG pathway of focal adhesion signaling pathway.

KEGG pathway	Enriched genes
Focal adhesion	COMP, PPP1R12B, LAMA4, ITGA9, MYLK, COL4A6, FLT4, TLN2, ITGA7, COL4A5, COL4A2, SHC2, PARVA, ROCK2, ITGA2, VEGFD, FN1, FLNA, PDGFRB, ITGA8, COL4A1

### Establishment of ROCK2-, PPP1R12B-, and VEGFD-related ceRNA networks

**FIGURE 6:**
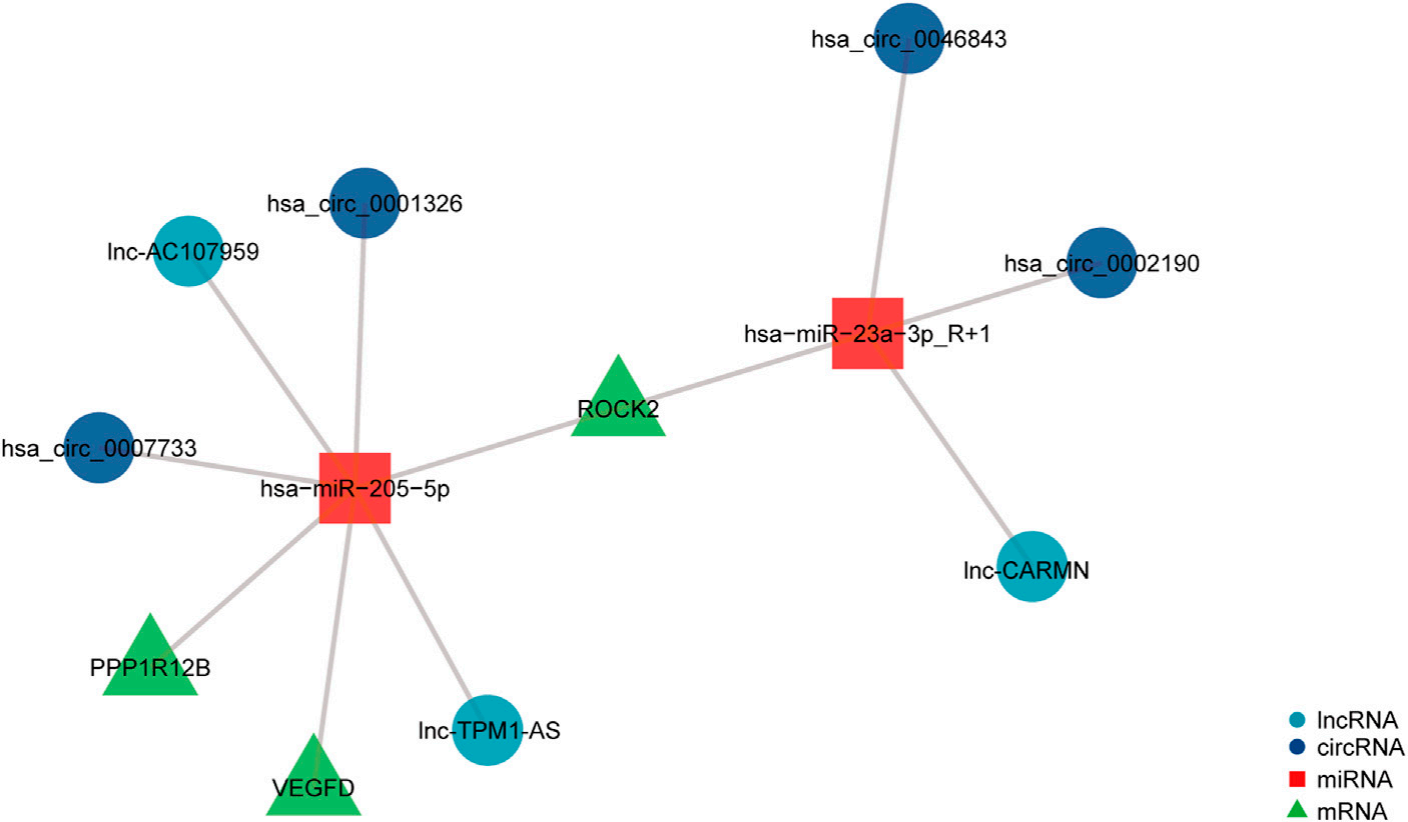
ceRNA network of differentially expressed circRNAs, lncRNAs, miRNAs, and mRNAs. The navy-blue circular nodes represent circRNAs, the light blue circular nodes represent lncRNAs, the square nodes represent miRNAs, and the triangular nodes represent mRNAs.

To investigate the potential regulatory involvement of DE ncRNAs, including circRNAs and lncRNAs, in targeting miRNAs in the focal adhesion signaling pathway in POP, we screened coexpressed genes from hub genes and then constructed and visualized the circRNA/lncRNA‒miRNA–mRNA ceRNA network using R package v3.6.3. We selected miRNAs and circRNAs with TargetScan scores >95 to obtain more reliable results. The core miRNAs were determined to be miR-23a-3p and miR-205-5p. Furthermore, hsa_circ_0002190, hsa_circ_0046843, and lnc-CARMN sponge miR-23a-3p with ROCK2 as the target gene. In addition, hsa_circ_0001326, hsa_circ_0007733, lnc-AC107959, and lnc-TPM1-AS regulate miR-205-5p, and hub genes such as ROCK2, PPP1R12B, and VEGFD were identified (Figure 6). Therefore, we have established ROCK2-, PPP1R12B-, and VEGFD-related ceRNA networks.

### Abnormal cytoskeleton of vaginal fibroblasts in patients with pelvic organ prolapse

**FIGURE 7:**
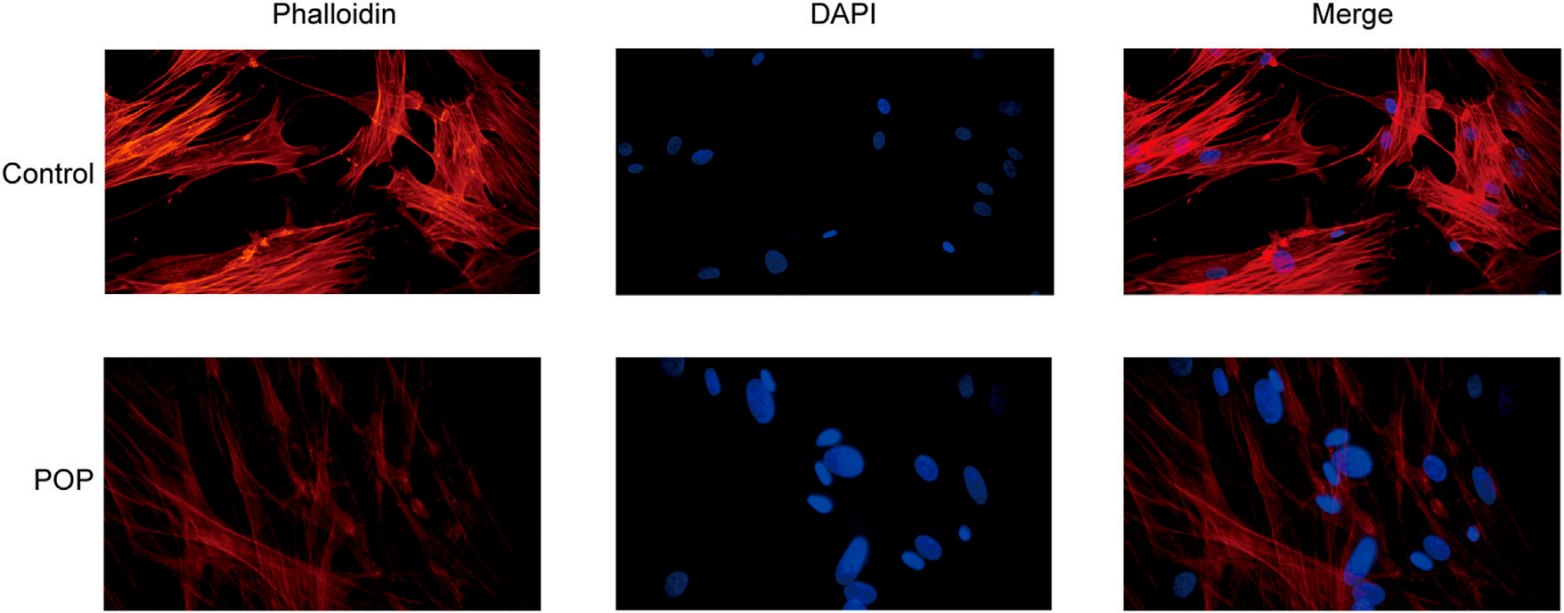
Cytoskeleton of fibroblasts from the POP and control groups. (400×).

The action of focal adhesion on the cytoskeleton is the main mechanism for the transmission of a mechanical stimulation signal from the cell membrane to the intracellular space. Therefore, to analyze the changes in the cytoskeleton of fibroblasts in patients with POP, F-actin was stained with phalloidin. The results revealed that actin fibroblast filaments from the anterior vaginal wall were disrupted (Figure 7). An abnormal actin phenotype was observed in the POP group, and this phenotype involved decomposition of the stretched component of the filament. Thus, the cytoskeleton of vaginal fibroblasts in patients with POP is abnormal.

## Discussion

POP is primarily characterized by the weakening of support tissue and can be caused by pelvic floor connective tissue extracellular matrix remodeling, activation of oxidative stress, genetic susceptibility, denervation of pelvic floor innervation, and reduced estrogen infiltration (Deng et al., 2021), however, the underlying mechanism, including the vital molecules and signaling pathways, is unknown. Furthermore, the role of regulatory ncRNAs in the pathogenesis of POP remains unclear. In this study, we performed whole transcriptome RNA sequencing to examine circRNAs, lncRNAs, miRNAs, and mRNAs in the anterior vaginal wall and identified 71 DECs, 76 DELs, 84 DEMs, and 931 DEGs. To test the accuracy of the whole transcriptomic data in this experiment, we chose 3 DECs, 3 DELs, 3 DEMs, and 3 DEGs at random for qRT‒PCR verification. The results showed the same trend between the two analyses and supported the high reliability of our data (R^2^ = 0.791).

GO and KEGG enrichment analyses were then performed to analyze the function of DEGs and demonstrated that the pathways were mainly associated with focal adhesion. *In vivo*, cells are in contact with the extracellular matrix, bind to ECM proteins through integrins on the membrane and recruit force-sensitive proteins, such as vinculin, talin, paxillin, and focal adhesion kinase, to form a focal adhesion, which further mediates the connection between the cell membrane and the cytoskeleton, is then converted into biochemical signals, and thereby ultimately affect gene expression (Hoffmann et al., 2019). Therefore, changes in the extracellular matrix play an important role in the regulation of cell adhesion, proliferation, migration, and apoptosis through mechanical signal transduction (Fiorentino et al., 2022). It is well known that remodeling of the ECM in the pelvic floor connective tissue is one of the reasons for the occurrence of POP (Deng et al., 2021), particularly the proportions of collagen Ⅰ and Ⅲ (Deprest et al., 2022). Ruiz-Zapata et al. (2016) demonstrated that matrices increase stiffness in postmenopausal women with POP compared with controls. Therefore, the mechanical microenvironment of fibroblasts surrounded by extracellular matrix is also altered. The “focal adhesion - cytoskeleton system” is considered the main mechanism through which force stimulation signals are transmitted through the cell membrane to the intracellular cell. In our study, we found that patients with POP have a disorder of the cytoskeleton structure compared with the control group. Therefore, the focal adhesion signaling pathway was the focus of this study. Kufaishi et al. (2016). revealed that the transcript levels of integrins (ITGA1, ITGA4, ITGAV, and ITGB1) are downregulated in control vaginal fibroblasts during mechanical stretching, whereas ITGA2, ITGA4, ITGA6, ITGB1, contactin (CNTN1), catenins (A1 and B1), and laminins (A3 and C1) are significantly upregulated in vaginal fibroblasts from POP patients. Cecati et al. (2018) demonstrated a significant upregulation of extracellular matrix protein 1 (ECM1) and integrin beta 3 (ITGB3) and downregulation of FBLN5 in the POP group by real-time PCR and PCR array. The upregulation of ECM1 avoids collagen degradation and extracellular matrix remodeling by inhibiting matrix metalloproteinase-9 (MMP-9) activity (Fujimoto et al., 2006; Rapisarda et al., 2017). Furthermore, ITGB3 could cause a significant increase in transforming growth factor beta 1 (TGF-β1) activity, which could stimulate fibroblasts to increase FBLN-5 expression (Asano et al., 2005). Therefore, the reduced expression of FBLN5 may be the starting point. The pelvic tissue then increases the expression of both ITGB3 and ECM1 to compensate for a lack of FBLN5. In our study, integrins (ITGA2, ITGA7, ITGA8, and ITGA9) also showed differences in the anterior vaginal wall between the control and POP groups.

We chose 14 DEGs as hub genes and studied the interactions of ncRNAs, including circRNAs, lncRNAs, and miRNAs, using the PPI network. This analysis showed that hsa_circ_0002190, hsa_circ_0046843, and lnc-CARMN sponge miR-23a-3p with ROCK2 as the target gene. Furthermore, hsa_circ_0001326, hsa_circ_0007733, lnc-AC107959, and lnc-TPM1-AS regulate miR-205-5p, and hub genes such as ROCK2, PPP1R12B, and VEGFD were identified. A previous study showed that hsa_circ_0002190 expression is decreased in gastric cancer and that hsa_circ_0002190 accumulates preferentially in the cytoplasm (Dong et al., 2021). By regulating KIT and LAMC3, lnc-AC107959 may play a role in the mechanism of non-muscle invasive bladder cancer (He et al., 2018). Our study found that miR-23a-3p regulates ROCK2 expression. miR-23a-3p plays a role in tumor pathology by regulating proliferation, invasion, and glycolysis (Shi et al., 2021). However, the biological role of miR-23a-3p in POP has not been investigated. We found that miR-23a-3p plays a regulatory role by targeting ROCK2. ROCK2 is a serine/threonine kinase that regulates actin-mediated cytoskeleton contractility and is a member of the AGC family of serine/threonine kinases (Weber and Herskowitz, 2021). ROCK2 is also targeted by miR-205-5p in our ceRNA network. miR-205-5p, similar to miR-23a-3p, is involved in modulating the proliferation and invasion of gastric cancer cells as part of the mechanism through which the lncRNA AFAP1-AS1 regulates AFAP1 (Dang et al., 2021). Furthermore, our findings indicate that PPP1R12B and VEGFD are involved in the regulation of miR-205-5p. Liang et al. (Tao et al., 2022) showed that inhibition of the PI3K/Akt signaling pathway can upregulate the expression of miR-205-5p and that miR-205-5p can inhibit the production of VEGF-A in breast cancer cells as well as tumor angiogenesis and metastasis. The present study found that miR-205-5p targets VEGFD to participate in the focal adhesion signaling pathway. We hypothesize that miR-205-5p inhibits fibroblast proliferation *via* the focal adhesion signaling pathway by targeting VEGFD because cell adhesion molecules also regulate angiogenesis *via* their involvement in cell proliferation, migration, and survival. More research is needed to confirm this theory. Furthermore, hsa_circ_0001326, hsa_circ_0007733, lnc-AC107959, and lnc-TPM1-AS sponge miR-205-5p. In preeclampsia, hsa_circ_0001326 increases IL16 expression to regulate proliferation, migration, invasion, and EMT (Liu et al., 2021). However, no information is available regarding the biological roles of hsa_circ_0007733, lnc-AC107959, and lnc-TPM1-AS. Even though many previous studies have investigated proliferation, apoptosis, and cytoskeleton morphology and confirmed that many molecules are associated with the pathological process in POP, they did not focus on the focal adhesion signaling pathway and did not construct a ceRNA network.

The current study provided a transcription database of POP and constructed a focal adhesion signaling pathway-related ceRNA network to uncover the pathogenesis of POP. Furthermore, this dataset can be used as a useful public resource for the identification of new biomarkers and may also provide new insights into elucidating the pathology of POP. However, this study has some limitations. First, the sample sizes of the control and POP groups were insufficient and need to be increased in future studies. Second, our findings are based on bioinformatics analysis, and more samples will be needed for confirmation via qRT–PCR, Western blot, and other methods. Furthermore, the different grades of POP may have an impact on whole transcriptome RNA sequencing. The whole transcriptome RNA sequencing results provide only a preliminary screening study result. Multiple experimental studies are needed to validate the regulatory mechanism of circRNA/lncRNA/miRNA/mRNA in POP.

In summary, we built a ceRNA network for the focal adhesion signaling pathway in POP pathogenesis, which included hsa_circ_0002190/hsa_circ_0046843/lnc-CARMN - miR-23a-3p - ROCK2 and hsa_circ_0001326/hsa_circ_0007733/lnc-AC107959/lnc-TPM1-AS - miR-205-5p - ROCK2/PPP1R12B/VEGFD. Furthermore, we discovered abnormal changes in the cytoskeleton, which are the final target of focal adhesion signaling events. This study not only contributes to the understanding of POP pathogenesis, but the findings can also be used to target molecular interventions for POP using drug-available gene databases.

## Data Availability

The datasets presented in this study can be found in online repositories. The names of the repository/repositories and accession number(s) can be found in the article/Supplementary Material
