# Supplementary material for: Construction of a focal adhesion signaling pathway-related ceRNA network in pelvic organ prolapse by transcriptome analysis
Source: Front Genet. 2022 Sep 13;13:996310. doi: 10.3389/fgene.2022.996310 (PMC9513229; doi:10.3389/fgene.2022.996310)
Supplement: Supplementary file 1 [file DataSheet1.docx]

Supplementary Material

# Supplementary Figures and Tables

## Supplementary Figures


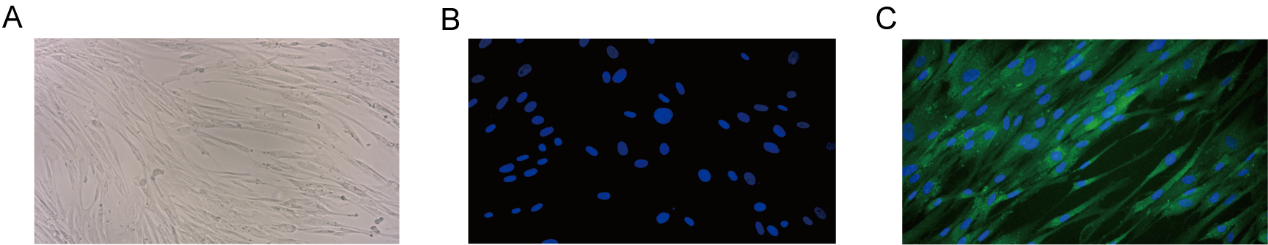


Supplementary Figure 1. Morphology and identification of fibroblasts. (A) Long spindle-shaped fibroblasts (200×). (B) Negative immunofluorescence staining for pan-cytokeratin (200×). (C) Positive immunofluorescence staining for vimentin (200×)

## Supplementary Tables

**Supplementary Table 1.** Demographic characteristics of participants

| No. | Age^a^ (years) | BMI^a^ (kg/m^2^) | Time since menopause^a^ | Gravidity | Vaginal birth | Previous surgical history | Hypertension | Diabetes mellitus | Immune disorders history | Position of prolapse |
| --- | --- | --- | --- | --- | --- | --- | --- | --- | --- | --- |
| POP 1 | 47 | 26.64 | 2 | 3 | 1 | Bilateral salpingectomy | No | No | No | Anterior vaginal wall |
| POP 2 | 65 | 22.03 | 18 | 2 | 2 | No | No | No | No | Anterior/ posterior vaginal wall+uterine |
| POP 3 | 71 | 24.34 | 19 | 7 | 3 | Cholecystectomy | No | No | No | Anterior vaginal wall+uterine |
| POP 4 | 77 | 21.09 | 24 | 4 | 3 | Bilateral tubal ligation | Yes | No | No | Anterior vaginal wall+uterine |
| POP 5 | 71 | 20.82 | 17 | 6 | 2 | No | No | Yes | No | Anterior vaginal wall+uterine |
| POP 6 | 66 | 21.88 | 11 | 3 | 1 | No | No | No | No | Anterior vaginal wall+uterine |
| Con 1 | 76 | 22.06 | 30 | 3 | 2 | No | Yes | Yes | No | - |
| Con 2 | 58 | 21.09 | 8 | 3 | 2 | No | No | No | No | - |
| Con 3 | 60 | 22.30 | 8 | 4 | 1 | No | No | No | No | - |
| Con 4 | 54 | 20.50 | 3 | 2 | 2 | No | No | No | No | - |
| Con 5 | 55 | 22.38 | 2 | 4 | 2 | Cholecystectomy | No | No | No | - |
| Con 6 | 63 | 21.48 | 17 | 2 | 1 | No | Yes | Yes | No | - |

BMI: Body mass index; POP: Pelvic organ prolaps; Con: Control; Immune disorder: asthma autoimmune diseases such as systemic lupus erythematosus, rheumatic disease, or osteoarthritis, etc.

^a^Two-tailed unpaired Student t-test was preformed to compare the age, BMI, and time since menopause between two groups. Age: *P* = 0.229; BMI: *P* = 0.732; Time since menopause: *P* = 0.375

**Suppenmentary Table 2.** Specific primer sequences for qRT-PCR.

| **Gene** |  | **Primer sequences** |
| --- | --- | --- |
| COL1A1 | Forword | GCCATCAAAGTCTTCTGCAACA |
|  | Reverse | GGAATCCATCGGTCATGCTCT |
| COL3A1 | Forword | CGAGCTTCCCAGAACATCACA |
|  | Reverse | TGCAACCATCCTCCAGAACTGT |
| ELN | Forword | GCAGGAGTTAAGCCCAAGG |
|  | Reverse | TGTAGGGCAGTCCATAGCCA |
| MMP2 | Forword | GTGGATGATGCCTTTGCTCG |
|  | Reverse | GGAGTCCGTCCTTACCGTCAA |
| MMP9 | Forword | GCCCTTCTACGGCCACTACTGT |
|  | Reverse | TTCCCATCCTTGAACAAATACAGC |
| TIMP2 | Forword | CTGTGACTTCATCGTGCCCTG |
|  | Reverse | GGAGGAGATGTAGCACGGGAT |
| VSIG10L | Forword | CTGAGGGCCAAGATTTGAGCC |
|  | Reverse | CAGACAGTTTGGTATGGGAGAC |
| KLF2 | Forword | CTACACCAAGAGTTCGCATCTG |
|  | Reverse | CCGTGTGCTTTCGGTAGTG |
| NID1 | Forword | CGGGGATGACTTCGTCTCTC |
|  | Reverse | GTGGTGACGTAGACTGCGT |
| hsa-miR-100-5p | Forword | AAGCACAACCCGTAGATCCGA |
| hsa-miR-126-5p | Forword | CTGCGCATTATTACTTTTGGTACGCG |
| hsa-miR-200c-3p | Forword | AAGTCGCTAATACTGCCGGGT |
| LINC02018 | Forword | TGGTGTCTTTGGCTGACAGA |
|  | Reverse | CGGCACGTACTTCCTACCAA |
| AC068790 | Forword | CTTCACCCTTCATGGACAGCCTTC |
|  | Reverse | GCTCACTGTGTACTCAGTCCTATGC |
| AL358115 | Forword | CCCAAGCCAGCTAAAGGAGC |
|  | Reverse | TCAGACCCTCCTCCTCAGGT |
| TJP1 | Forword | AAAGAGAAAGGTGAAACACTGC |
|  | Reverse | TTTTAGAGCAAAAGACCAACCG |
| FTO | Forword | GTTCACAACCTCGGTTTAGTTC |
|  | Reverse | CATCATCATTGTCCACATCGTC |
| MAML2 | Forword | GGAACTGTTCAATGAACTGACC |
|  | Reverse | GCTTTATGGTGGCATTGATCAT |
| FLT4 | Forword | AATCTGGCACCACACCTTCTACAA |
|  | Reverse | GGATAGCACAGCCTGGATAGCAA |
| COL4A2 | Forword | TGCACGAGGTACATGCCAAC |
|  | Reverse | GCTGCTCAAAGTCTCTCACGAA |
| BCL2 | Forword | TTATGCACTGCCTAAAGAGGAGC |
|  | Reverse | CCCTTAACTCCGTAGAAACCAAG |
| LAMA4 | Forword | GGTGGGGTCATGTGTGTGG |
|  | Reverse | CGGTTCAGGTACTCAGTCATCC |
| TGFBR2 | Forword | CCAGTGTAGGAATTGCTTACGC |
|  | Reverse | TAACCGCAGGTCATCAGTCAG |
| FYN | Forword | GTAGCTCTGATGAGTGCAATGAC |
|  | Reverse | CAGATATGGCAACTCCCAGTG |
| PRKCB | Forword | AGCCCCACGTTTTGTGACC |
|  | Reverse | GCTGGGAACATTCATCACGC |
| RYR2 | Forword | GGCAGCCCAAGGGTATCTC |
|  | Reverse | ACACAGCGCCACCTTCATAAT |
| ATP1A2 | Forword | CACCACCGAAGATCAGTCTGG |
|  | Reverse | CGCTTAGACACGGAGATGTTC |
| PIP5K1B | Forword | CTGGGAATAGGATACACAGTGGG |
|  | Reverse | GCTGGGTAGGAACACACTTTC |
| β-actin | Forword | AATCTGGCACCACACCTTCTACAA |
|  | Reverse | GGATAGCACAGCCTGGATAGCAA |
